# Maternal *FNDC5* and *ADIPOQ* Genetic Variants as Potential Modulators of Susceptibility to Spontaneous Preterm Birth

**DOI:** 10.3390/ijms27219945

**Published:** 2026-11-06

**Authors:** Mirta Kadivnik, Patricija Zlatunić, Mia Drinić, Rajna Hercog, Kristina Kralik, Jasenka Wagner

**Affiliations:** 1 Department of Obstetrics and Gynecology, Faculty of Medicine, University of Osijek, 31000 Osijek, Croatia; mirta.kadivnik@kbco.hr; 2 Department of Medical Biology and Genetics, Faculty of Medicine, University of Osijek, 31000 Osijek, Croatia; 3 Department of Medical Statistics and Informatics, Faculty of Medicine, University of Osijek, 31000 Osijek, Croatia

**Keywords:** preterm birth, *ADIPOQ*, adiponectin, *FNDC5*, irisin, rs266729, rs1746661, maternal genetic variation, SNP

## Abstract

Preterm birth (PTB) is a complex and heterogeneous pregnancy outcome in which maternal metabolic and endocrine pathways may contribute to susceptibility to preterm delivery. This study investigated whether two maternal genetic variants involved in metabolic signaling, *ADIPOQ* rs266729 and *FNDC5* rs1746661, are associated with preterm birth (PTB) in a Croatian population. This case–control study included 401 women: 200 with PTB and 201 with term delivery. Maternal genomic DNA was isolated from peripheral blood, and both variants were genotyped using TaqMan-based real-time PCR. Genotype and allele distributions, inheritance models, allelic combinations, logistic regression, and gene–gene interaction were analyzed. Neither variant was significantly associated with overall PTB, and no significant gene–gene interaction was observed (*p* = 0.18). In early PTB, only a borderline, non-significant tendency toward a higher *ADIPOQ* rs266729 G allele frequency was found (39% vs. 27%; OR 1.74, 95% CI 0.99–3.04; *p* = 0.05), which was not supported by genotype, inheritance-model, or regression analyses. The G–T allelic combination was more frequent in early PTB (13.6% vs. 4.6%; *p* = 0.03) but not after Benjamini–Hochberg correction (adjusted *p* = 0.09). No significant association between the investigated variants and PTB was demonstrated. The borderline findings in early PTB are hypothesis-generating and require replication in larger cohorts.

## 1. Introduction

Preterm birth (PTB), defined as birth before 37 completed weeks of gestation, remains one of the leading causes of neonatal morbidity and mortality worldwide [1]. Preterm birth (PTB), which includes spontaneous preterm labor and preterm prelabor rupture of membranes, represents a complex, biologically heterogeneous syndrome resulting from the interaction of genetic, inflammatory, metabolic, endocrine, placental, and environmental factors [2]. Despite substantial advances in understanding the mechanisms involved in the initiation of human parturition, the molecular determinants responsible for the premature activation of these pathways remain incompletely understood.

Growing evidence suggests that maternal metabolic and endocrine signaling contributes to the regulation of pregnancy and may influence the timing of parturition. Adipose tissue is an active endocrine organ that produces a broad range of adipokines involved in energy homeostasis, insulin sensitivity, inflammation, vascular function, and immune regulation [3]. These metabolic and inflammatory pathways are closely interconnected with placental function and maternal adaptation to pregnancy, providing a biological rationale for investigating maternal metabolic signaling as a potential determinant of gestational duration.

Adiponectin, encoded by the *ADIPOQ* gene, is one of the most abundant adipose-derived hormones and has predominantly insulin-sensitizing and anti-inflammatory properties. Adiponectin regulates glucose and lipid metabolism through its receptors, AdipoR1 and AdipoR2, and participates in pathways involved in metabolic homeostasis and inflammatory regulation [4]. Unlike many other adipokines, circulating adiponectin concentrations are generally inversely associated with adiposity and are altered in obesity and insulin resistance [4,5]. Because metabolic adaptation and tightly regulated inflammatory responses are essential for normal placental function and pregnancy maintenance, the adiponectin system represents a biologically plausible pathway through which maternal genetic variation could influence pregnancy outcomes.

Evidence directly linking adiponectin to the mechanisms of spontaneous preterm parturition has also been reported. Mazaki-Tovi et al. demonstrated dysregulation of maternal serum adiponectin in women with preterm labor, particularly in the presence of intra-amniotic infection or inflammation [6]. Furthermore, adiponectin has been identified as a physiological component of amniotic fluid, with concentrations varying according to gestational age and increasing in the presence of intra-amniotic infection/inflammation in women with spontaneous preterm labor [7]. These findings are of particular interest because intrauterine infection and inflammation represent important pathways leading to spontaneous preterm delivery.

Genetic variation within the *ADIPOQ* gene may contribute to interindividual differences in adiponectin expression and metabolic phenotypes. The *ADIPOQ* rs266729 (−11377C>G) single-nucleotide polymorphism (SNP) is a common promoter polymorphism that has been extensively investigated in relation to obesity, insulin resistance, type 2 diabetes, and other metabolic phenotypes [4,5]. Importantly, rs266729 has also been studied in pregnancy. Beltcheva et al. reported a significant association between rs266729 and gestational diabetes mellitus (GDM) in a cohort of 394 women, with the minor G allele being more frequent among controls and suggested to have a protective effect against GDM [8]. A subsequent meta-analysis including 12 studies and more than 8000 participants confirmed that rs266729 is associated with GDM susceptibility, although the direction and magnitude of the association differ among populations, emphasizing the potential influence of genetic background on this relationship [9].

The relationship between maternal metabolic dysfunction, adiponectin signaling, and preterm birth provides additional rationale for investigating *ADIPOQ* SNP rs266729 in PTB. In women with GDM, Mierzyński et al. reported lower maternal adiponectin concentrations among those who subsequently delivered preterm, supporting a potential link between altered adipokine signaling, metabolic dysfunction, and preterm delivery [10]. Although these observations do not establish a direct causal relationship between *ADIPOQ* SNP rs266729 and PTB, they support the hypothesis that genetic differences affecting adiponectin-related pathways may contribute to maternal susceptibility to adverse pregnancy outcomes.

In parallel with adiponectin, the fibronectin type III domain-containing protein 5 (*FNDC5*) gene encodes the precursor of irisin, a myokine/adipomyokine involved in energy metabolism, glucose homeostasis, lipid metabolism, and cellular signaling. *FNDC5* is expressed in skeletal muscle and other tissues, and its expressed product is proteolytically cleaved to release irisin, which has been implicated in the regulation of energy expenditure and metabolic homeostasis [11,12]. Although the biological functions of *FNDC5*/irisin remain incompletely characterized, increasing evidence indicates that this signaling system may also be relevant to reproductive biology and pregnancy.

The *FNDC5*/irisin pathway may be particularly relevant to pregnancy because irisin has been detected in maternal circulation and umbilical cord blood, and its concentrations have been investigated in relation to pregnancy complications and fetal growth [12,13]. Pavlova et al. specifically examined maternal and cord blood irisin concentrations in women with spontaneous preterm and term deliveries. Although maternal plasma irisin concentrations did not differ significantly between the groups, umbilical cord blood irisin concentrations were associated with PTB in univariable analysis, suggesting a possible role of the *FNDC5*/irisin system in fetal–placental adaptation and preterm birth [13].

The potential contribution of *FNDC5* genetic variation to PTB has also been investigated. Salem et al. examined maternal and neonatal *FNDC5* rs726344 and rs1746661 polymorphisms in an Israeli cohort comprising 315 mothers and 161 neonates. The maternal rs726344 GG genotype was associated with a higher likelihood of term delivery, whereas no significant association between rs1746661 and PTB was identified [14]. Although these findings do not establish rs1746661 as a PTB susceptibility variant, they provide an important precedent for investigating *FNDC5* genetic variation in independent populations and highlight the possibility of population-specific genetic effects.

Experimental evidence further supports the potential role of *FNDC5*/irisin signaling in placental biology. A recent study using an *FNDC5*-deficient mouse model demonstrated alterations in placental development, maternal body weight, and fetal–placental growth, suggesting that *FNDC5* signaling may contribute to the regulation of placental structure and fetal–placental adaptation [15]. These findings provide additional biological plausibility for investigating maternal *FNDC5* genetic variation in relation to pregnancy duration.

Taken together, the adiponectin and *FNDC5*/irisin systems represent metabolically active endocrine pathways that may influence inflammatory regulation, energy homeostasis, placental function, and maternal–fetal adaptation. However, the contribution of maternal genetic variation within these pathways to spontaneous preterm birth remains insufficiently characterized. *ADIPOQ* rs266729 has been extensively investigated in metabolic and pregnancy-related phenotypes but has not been adequately evaluated as a maternal genetic determinant of PTB. Similarly, evidence concerning *FNDC5* rs1746661 and PTB remains limited, with the available study reporting no significant association.

Therefore, the aim of the present study was to investigate the association of two maternal metabolic gene variants, *FNDC5* rs1746661 and *ADIPOQ* rs266729, with susceptibility to spontaneous preterm birth in a Croatian population. We further evaluated genotype and allele distributions under different genetic inheritance models to determine whether these variants contribute to maternal genetic susceptibility to spontaneous preterm delivery.

## 2. Results

### 2.1. Clinical and Demographic Characteristics of the Study Population

The study population comprised 401 women, including 201 with term birth and 200 with spontaneous preterm birth. The demographic and clinical characteristics of the study population are presented in Table 1. Maternal age and BMI did not differ significantly between women with term and preterm birth. Similarly, no significant differences were observed in coffee consumption, smoking, urinary tract infection, or positive cervical swab results.

In contrast, pregnancy complications were more frequently reported among those with PTB than among women with term birth (75.5% vs. 51.7%, *p* < 0.001). Vaginal bleeding during pregnancy was also significantly more frequent in the PTB group (23.0% vs. 7.0%, *p* < 0.001). PROM and a family history of preterm birth were significantly more common among women with PTB. Because a personal or family history of PTB was an exclusion criterion for the control group, the differences in these variables reflect the study design rather than an independent finding. The median gestational age was significantly lower in the PTB group (34 + 6 weeks vs. 39 + 4 weeks, *p* < 0.001), and cesarean delivery was more frequent in women with PTB (29.0% vs. 2.5%, *p* < 0.001). As expected, birth weight was significantly lower among infants born preterm (median 2425 g vs. 3450 g, *p* < 0.001).

### 2.2. Distribution of ADIPOQ rs266729 and FNDC5 rs1746661 Genotypes

Genotype data were available for 199 women with term birth and 199 women with PTB for *ADIPOQ* rs266729 and for 198 women with term birth and 195 women with PTB for *FNDC5* rs1746661. Therefore, genotype-based analyses were performed using the available valid genotype data for each variant. Genotype and allele distributions, inheritance models, and Hardy–Weinberg equilibrium were assessed separately for each variant (Table 2).

The genotype and allele distributions of *ADIPOQ* rs266729 and *FNDC5* rs1746661 in women with term and preterm birth are shown in Table 2. Both variants were in Hardy–Weinberg equilibrium in both the term and PTB groups.

For *ADIPOQ* rs266729, the C/C genotype was present in 54.3% of women with term birth and 46.7% of women with PTB, whereas the G/G genotype was observed in 7.5% and 10.6% of women respectively. The differences in genotype distributions and allele frequencies between the two groups were not statistically significant. None of the evaluated inheritance models demonstrated a significant association between rs266729 and overall PTB.

Similarly, no significant differences were observed in the distribution of *FNDC5* rs1746661 genotypes or alleles between women with term and preterm birth. The G/G genotype was the most frequent in both groups, and none of the dominant, recessive, or overdominant inheritance models showed a statistically significant association with PTB.

### 2.3. Combined Two-Locus Allelic Analysis

A combined two-locus allelic analysis was performed to assess whether combinations of *ADIPOQ* rs266729 and *FNDC5* rs1746661 alleles were associated with overall PTB (Table 3). The C–G allelic combination was the most frequent in both groups and was used as the reference. The frequencies of the G–G, C–T, and G–T allelic combinations did not differ significantly between women with term and preterm birth. The global allelic combination association was not statistically significant (*p* = 0.10).

### 2.4. Bivariate Logistic Regression for Overall PTB

To further evaluate the potential contribution of the investigated variants to overall PTB, bivariate logistic regression analysis was performed (Table 4). Neither *ADIPOQ* rs266729 nor *FNDC5* rs1746661 was significantly associated with the odds of PTB. The ORs for carriage of the *ADIPOQ* rs266729 G allele (OR 1.35, 95% CI 0.92–2.01; *p* = 0.13) and of the *FNDC5* rs1746661 T allele (OR 0.75, 95% CI 0.49–1.13; *p* = 0.17) were not statistically significant.

### 2.5. Association of the Investigated Variants with Early PTB

Because PTB represents a heterogeneous phenotype, we further analyzed the distribution of the investigated variants by gestational age. Table 5 presents the genotype and allele distributions of *ADIPOQ* rs266729 and *FNDC5* rs1746661 in women with early PTB and term birth. The early PTB subgroup included 31 women. For *FNDC5* rs1746661, genotype data were available for 29 of the 31 women with early PTB.

For *ADIPOQ* rs266729, there was a borderline tendency toward a higher G allele frequency among women with early PTB than among women with term birth (39% vs. 27%; OR 1.74, 95% CI 0.99–3.04; *p* = 0.05), with a confidence interval that included 1. The overall genotype distribution did not differ significantly between the groups (*p* = 0.17); the G/G genotype was numerically more frequent in the early PTB group (16.1% vs. 7.5%), but this difference was not statistically significant (OR 3.00, 95% CI 0.93–9.71). None of the evaluated inheritance models reached statistical significance.

In contrast, *FNDC5* rs1746661 did not differ significantly between women with early PTB and term birth. The G allele was observed in 78% of women with early PTB and 79% of women with term birth (OR for the T allele = 1.07, 95% CI, 0.55–2.08; *p* = 0.83), and none of the evaluated inheritance models showed a significant association.

### 2.6. Association of ADIPOQ rs266729 and FNDC5 rs1746661 with Other Gestational-Age-Specific PTB Phenotypes

To further assess whether the observed findings varied by gestational age at delivery, we also analyzed the associations of *ADIPOQ* rs266729 and *FNDC5* rs1746661 with extremely early and late PTB. In the extremely early PTB subgroup *(n* = 17), neither rs266729 nor rs1746661 showed a statistically significant difference in genotype or allele distribution compared with term birth (Table S1, Supplementary Materials). Similarly, neither variant was significantly associated with extremely early PTB under any of the evaluated inheritance models. Combined Two-Locus Allelic analysis also revealed no significant association between rs266729–rs1746661 allelic combinations and extremely early PTB (Table S2, Supplementary Materials), and neither variant was significantly associated with extremely early PTB in bivariate logistic regression analysis (Table S3, Supplementary Materials).

In the late PTB subgroup, neither *ADIPOQ* rs266729 nor *FNDC5* rs1746661 was significantly associated with late PTB in genotype, allele, or inheritance-model analyses (Table S4, Supplementary Materials). Likewise, Combined Two-Locus Allelic analysis did not demonstrate a significant association between rs266729–rs1746661 allelic combinations and late PTB (Table S5, Supplementary Materials), and neither variant was significantly associated with late PTB in bivariate logistic regression analysis (Table S6, Supplementary Materials).

Thus, in this cohort, the borderline tendency for *ADIPOQ* rs266729 was observed only in the early PTB subgroup and not in the extremely early or late PTB subgroups.

### 2.7. Combined Two-Locus Allelic Analysis in Early PTB

We next examined the combined distribution of *ADIPOQ* rs266729 and *FNDC5* rs1746661 allelic combinations in women with early PTB (Table 6). The C–G allelic combination was the most frequent allelic combination and served as the reference. The G–T allelic combination was more common among women with early PTB than among women with term birth (13.6% vs. 4.6%) and was associated with higher odds of early PTB in the unadjusted analysis (OR = 2.83, 95% CI, 1.11–7.22; *p* = 0.03). However, this association was no longer statistically significant after Benjamini–Hochberg correction (adjusted *p* = 0.09). The global allelic combination association was not statistically significant (*p* = 0.10).

### 2.8. Genotype-Based Interaction Analysis of ADIPOQ and FNDC5

An additional binary logistic regression analysis was performed to assess the interaction between *ADIPOQ* rs266729 and *FNDC5* rs1746661 under dominant inheritance models. The analysis included 393 women with complete genotype data. No statistically significant interaction was observed (OR = 1.78; 95% CI, 0.77–4.13; *p* = 0.18). The overall model was not statistically significant (Chi-square test = 6.03; df = 3, *p* = 0.11; Nagelkerke R^2^ = 0.020). Given the limited number of women with early PTB, the interaction analysis was not performed in the gestational-age subgroups.

### 2.9. Bivariate Logistic Regression for Early PTB

Finally, a bivariate logistic regression analysis was performed to assess the independent contribution of the investigated variants to early PTB (Table 7). Neither *ADIPOQ* rs266729 nor *FNDC5* rs1746661 was significantly associated with early PTB in the regression model. The ORs for carriage of the *ADIPOQ* G allele (OR 1.68, 95% CI 0.79–3.56; *p* = 0.18) and of the *FNDC5* T allele (OR 1.14, 95% CI 0.52–2.49; *p* = 0.74) were not statistically significant.

The sensitivity power analysis indicated minimum detectable odds ratios of approximately 1.76 for overall PTB, 3.06 for early PTB, and 4.53 for extremely early PTB, based on the dominant inheritance model of *ADIPOQ* rs266729, 80% statistical power, and a two-sided significance level of 0.05.

## 3. Discussion

Preterm birth (PTB) is a complex and heterogeneous pregnancy outcome in which genetic susceptibility interacts with metabolic, inflammatory, endocrine, placental, and environmental factors. In the present study, we examined two maternal genetic variants involved in metabolic signaling, *ADIPOQ* rs266729 and *FNDC5* rs1746661, in relation to spontaneous PTB. The main finding was that neither variant was significantly associated with overall PTB. However, when PTB was stratified by gestational age, only a borderline, non-significant tendency toward a higher *ADIPOQ* rs266729 G allele frequency was observed in early PTB, whereas no such tendency was observed for extremely early or late PTB. In addition, the G–T allelic combination combining *ADIPOQ* rs266729 and *FNDC5* rs1746661 was more frequent among women with early PTB in the unadjusted analysis, although this association did not remain statistically significant after correction for multiple comparisons. In contrast, *FNDC5* rs1746661 showed no significant association with PTB in any of the analyzed phenotypes. Likewise, no significant gene–gene interaction between the two variants was observed.

These findings raise the possibility that maternal genetic variation in metabolic signaling pathways may be related to gestational-age-specific PTB phenotypes. However, the absence of statistically significant associations in the overall analysis and after correction for multiple comparisons indicates that these observations are exploratory and require confirmation in larger cohorts.

### 3.1. ADIPOQ rs266729 and Early Preterm Birth

Adiponectin is an adipocyte-derived hormone with important metabolic and immunomodulatory functions. Through its receptors, AdipoR1 and AdipoR2, adiponectin regulates glucose and lipid metabolism, insulin sensitivity, inflammatory responses, and cellular energy homeostasis [4,5]. Increasing evidence also indicates that adiponectin signaling may participate directly in reproductive physiology and the maintenance of pregnancy.

One particularly relevant observation is that adiponectin may influence myometrial activity. Vyas et al. demonstrated that human and mouse myometrium express adiponectin receptors and that adiponectin and pharmacologic AdipoR activation inhibit myometrial contractions. Their findings further implicated AMPK signaling in this process, suggesting that adiponectin may represent a molecular link between maternal metabolic status and uterine quiescence [16]. This observation is of particular interest in the context of spontaneous PTB because premature activation of pathways leading to myometrial contractility represents one of the central mechanisms of preterm parturition.

Clinical studies have also reported altered adiponectin concentrations in association with spontaneous preterm labor. Mazaki-Tovi et al. demonstrated dysregulation of maternal serum adiponectin in women with preterm labor, particularly in the presence of intra-amniotic infection or inflammation [6]. Adiponectin has also been detected in amniotic fluid, where its concentrations vary with gestational age and are associated with intra-amniotic infection and inflammation [7]. Together, these findings suggest that adiponectin may participate at the interface between metabolic and inflammatory pathways involved in the initiation of preterm parturition.

In this context, our findings concerning *ADIPOQ* rs266729 are of interest. The rs266729 C>G variant is in the promoter region of *ADIPOQ* and has previously been investigated in relation to adiponectin concentrations and metabolic phenotypes [4,5]. Functional studies of the *ADIPOQ* promoter have demonstrated that promoter variation can affect transcriptional activity and adiponectin regulation, providing biological plausibility for considering regulatory *ADIPOQ* variants as potential modifiers of metabolic phenotypes [17]. Furthermore, a meta-analysis of *ADIPOQ* polymorphisms reported an association of rs266729 with circulating adiponectin concentrations, although the magnitude and direction of the associations varied among populations [18].

Regulatory annotation provides only partial support for a functional role of this variant. In RegulomeDB, rs266729 has a rank of 1f, indicating eQTL evidence together with transcription factor binding or a DNase I hypersensitivity peak [19,20], and the C>G substitution has been predicted to create a CpG site, introducing a potential methylation site within the *ADIPOQ* promoter [20]. However, rs266729 is not reported as a significant eQTL for *ADIPOQ* in any tissue in the Genotype-Tissue Expression (GTEx) portal [21]. Experimental data are also inconsistent: in reporter-gene assays, substitution of minor alleles at *ADIPOQ* promoter variants, including rs266729, altered promoter activity [17], whereas another study found no significant allelic effect of rs266729 on promoter activity [22]. Moreover, the direction of the association between the G allele and circulating adiponectin concentrations differs among populations [18,20]. Therefore, whether the G allele increases or decreases adiponectin expression in pregnant women remains to be established.

Despite this biological plausibility, we did not observe a significant association between rs266729 and overall spontaneous PTB. Genotype and allele distributions were comparable between women with PTB and term controls, and none of the evaluated inheritance models demonstrated a statistically significant association. This finding is important because it argues against a strong or generalizable effect of rs266729 on susceptibility to spontaneous PTB in our population.

After stratification by gestational age, only a borderline tendency was observed among women with early PTB, in whom the G allele was more frequent than among term controls (39% vs. 27%), corresponding to an OR of 1.74 (95% CI, 0.99–3.04; *p* = 0.05). The GG genotype was numerically more frequent in the early PTB group (16.1% vs. 7.5%), but this difference was not statistically significant (OR 3.00, 95% CI 0.93–9.71), and the overall genotype distribution did not differ between the groups (*p* = 0.17). Although these findings are suggestive, the confidence intervals are wide, and neither the dominant model nor the bivariate regression analysis reached statistical significance. In the regression model, the OR for G allele carriage (1.68, 95% CI 0.79–3.56; *p* = 0.18) was not statistically significant. Given the small number of women with early PTB and the multiple genetic comparisons performed, these nominal associations should be interpreted cautiously and regarded as exploratory. Therefore, the present data should not be interpreted as demonstrating a confirmed association between rs266729 and early PTB.

Nevertheless, the phenotype-specific pattern of this tendency may be informative for future studies. PTB is increasingly recognized as a heterogeneous syndrome rather than a single biological entity [23,24]. The mechanisms contributing to delivery at very early gestational ages may differ from those involved in late preterm delivery, with varying contributions from placental dysfunction, inflammation, infection, maternal metabolic adaptation, and other pathways [24,25]. A modest genetic effect could therefore be diluted when all spontaneous PTBs are combined into a single phenotype [25].

The observation that the rs266729 signal was present in early PTB but not in late PTB is therefore compatible with a possible gestational-age-dependent effect. Importantly, however, this interpretation remains speculative. Neither the extremely early PTB subgroup nor the late PTB subgroup showed evidence of an association with rs266729. In the extremely early PTB group, the G allele was less frequent than in term controls, and genotype comparisons were not significant. Similarly, no significant association was observed for late PTB.

One possible explanation for the observed early-PTB signal is that altered adiponectin signaling could simultaneously influence several processes relevant to pregnancy maintenance. Reduced adiponectin signaling has been associated with impaired metabolic homeostasis and increased inflammatory activity, while experimental data suggest that adiponectin can inhibit myometrial contractility through AdipoR-associated signaling [16]. In addition, adiponectin acts directly on the placenta, where it inhibits insulin signaling and insulin-stimulated amino acid transport in primary human trophoblasts [26], linking maternal adiponectin to placental nutrient transport and fetal growth. Therefore, a regulatory variant potentially affecting adiponectin expression could theoretically influence the threshold at which the uterus transitions from quiescence to active labor. However, this mechanism cannot be directly established in our study because circulating adiponectin concentrations and *ADIPOQ* expression were not measured. This proposed mechanism is therefore hypothetical and should be tested in studies combining genotyping with measurements of circulating adiponectin and placental and myometrial *ADIPOQ*/AdipoR expressions.

The interpretation of rs266729 is further complicated by the considerable population heterogeneity reported for this variant. Polymorphism has been associated with gestational diabetes mellitus (GDM), but the direction and magnitude of its association have varied among populations [8,9]. Beltcheva et al. reported that the G allele was more frequent among controls than among women with GDM, suggesting a possible protective effect in their population [8]. These findings, together with meta-analytic evidence of heterogeneous effects of rs266729 [18], indicate that the biological consequences of this variant may depend on population-specific genetic background and environmental or metabolic context.

This issue may be particularly relevant when considering PTB, because maternal metabolic phenotype, BMI, insulin sensitivity, and inflammation can all influence adiponectin biology. Mierzyński et al. reported lower maternal adiponectin concentrations in women with GDM who subsequently delivered preterm, supporting an association between altered adipokine signaling and preterm delivery, although the contribution of GDM itself could not be completely separated from the effect of adiponectin [10]. Our study differs from these investigations because we evaluated maternal genotype rather than circulating adiponectin concentration. Thus, our findings should be viewed as complementary rather than directly comparable to biomarker studies.

### 3.2. FNDC5 rs1746661 and Spontaneous Preterm Birth

The second genetic candidate investigated in this study was *FNDC5* rs1746661, located in the gene encoding the precursor of irisin. Irisin is a myokine/adipomyokine involved in energy metabolism, glucose homeostasis, lipid metabolism, and cellular signaling [15,27]. Growing evidence suggests that the *FNDC5*/irisin system also participates in placental biology and maternal–fetal adaptation [28,29,30].

Experimental studies provide a strong biological rationale for investigating *FNDC5* in pregnancy. Drewlo et al. demonstrated that irisin promotes trophoblast differentiation through activation of AMPK, accompanied by changes in integrin expression and increased trophoblast invasiveness [28]. These processes are essential components of normal placental development and function. In addition, Kohan-Ghadr et al. showed that irisin protects human placental cells from oxidative stress and apoptosis through activation of the Akt signaling pathway [29]. Thus, although the precise physiological role of irisin during pregnancy remains incompletely understood, current experimental evidence supports its involvement in placental cellular function, differentiation, and stress responses.

More recently, this biological relevance has been strengthened by an experimental study using an *FNDC5*-deficient mouse model. Nandi et al. reported altered placental architecture, reduced expression of labyrinth-associated markers and glucose transporter genes, and changes in vascular and fetal–placental parameters in *FNDC5*-deficient animals [15]. Importantly, *FNDC5* deficiency did not result in pregnancy failure but was associated with an altered placental phenotype and maternal–placental adaptation. These findings suggest that *FNDC5* may influence pregnancy primarily through modulation of placental function rather than acting as a direct determinant of pregnancy termination.

Despite this biological plausibility, our results provide no evidence that maternal *FNDC5* rs1746661 is associated with spontaneous PTB. In the overall comparison, the G allele was present in 79% of term controls and 83% of women with PTB, and the corresponding OR for the T allele was 0.78 (95% CI, 0.55–1.12; *p* = 0.18). None of the dominant, recessive, or overdominant models demonstrated a significant association.

This finding is consistent with the limited existing genetic literature. Salem et al. investigated maternal and neonatal *FNDC5* rs726344 and rs1746661 polymorphisms in an Israeli cohort and reported an association between maternal rs726344 genotype and PTB, whereas rs1746661 was not significantly associated with PTB [14]. Their findings suggested that the association with PTB may be variant-specific rather than reflecting a generalized effect of *FNDC5* genetic variation. Our results in an independent Croatian population are consistent with the absence of an association for rs1746661.

Pavlova et al. further investigated maternal and cord blood irisin concentrations together with several *FNDC5* polymorphisms in women with spontaneous PTB and term delivery [13]. Although maternal plasma irisin concentrations did not differ significantly between the groups, cord blood irisin showed an association with PTB in univariable analysis. Their findings suggest that the fetal–placental compartment may be more informative than maternal circulating irisin alone for understanding the role of this pathway in PTB. The absence of a maternal genetic association in our study is therefore compatible with a model in which *FNDC5*/irisin participates in placental biology without the investigated common variant exerting a sufficiently large effect on maternal PTB susceptibility.

Interestingly, the lack of association remained evident when PTB was stratified according to gestational age. In early PTB, the *FNDC5* rs1746661 allele distribution was similar to that of term controls, and the T allele yielded an OR of 1.07 (95% CI, 0.55–2.08; *p* = 0.83). No association was identified in extremely early or late PTB either. These findings argue against a major effect of rs1746661 on gestational timing in our cohort.

The discrepancy between the strong biological rationale for the *FNDC5*/irisin pathway and the absence of an association for rs1746661 illustrates an important principle in candidate-gene studies: biological relevance of a gene or pathway does not necessarily imply that every common genetic variant within that gene has a measurable phenotypic effect [31,32]. The functional consequences of a particular SNP depend on its genomic position, regulatory context, linkage disequilibrium with functional variants, and interaction with environmental and physiological factors [32]. Consequently, the absence of an association for rs1746661 does not exclude the role of *FNDC5*/irisin in PTB biology [14]. Notably, although rs1746661 is located in intron 2 of *FNDC5*, it shows strong regulatory annotation in RegulomeDB (rank 1b; probability score 0.97), based mainly on data from vascular tissue [19]. Moreover, in the GTEx portal, rs1746661 is a strong eQTL for *FNDC5* in skeletal muscle, the main source of circulating irisin (*p* = 1.9 × 10^−57^; normalized effect size 0.42 for the T allele) [21]. Therefore, the absence of an association is unlikely to reflect a lack of regulatory potential of this variant; rather, its regulatory effects may be tissue-specific and may not involve the placental or myometrial tissues relevant to parturition, or the effect on PTB susceptibility may be too small to be detected in our cohort. This interpretation is consistent with the observation that maternal plasma irisin concentrations did not differ between women with spontaneous preterm and term delivery [13].

### 3.3. Combined Analysis of ADIPOQ and FNDC5

The simultaneous investigation of *ADIPOQ* and *FNDC5* was motivated by the convergence of adiponectin and irisin signaling on several specific pathways relevant to pregnancy. First, both hormones activate AMP-activated protein kinase (AMPK): adiponectin through its receptors AdipoR1 and AdipoR2 [4], and irisin through integrin-mediated signaling [11]. AMPK signaling is directly relevant to parturition and placentation, because adiponectin-induced AMPK activation inhibits myometrial contractility [16], whereas irisin-induced AMPK activation promotes trophoblast differentiation and invasiveness [28]. Second, both hormones improve insulin sensitivity and regulate glucose and lipid metabolism, processes that are central to maternal metabolic adaptation to pregnancy [4,11,12]. Third, both exert anti-inflammatory and cytoprotective effects; adiponectin attenuates inflammatory signaling [4,5]; and irisin protects placental cells from oxidative stress and apoptosis [29]. Finally, both act directly on placental tissue, where adiponectin modulates trophoblast insulin signaling and nutrient transport [26], and *FNDC5*/irisin influences trophoblast differentiation and labyrinth development [15,28,30]. Variants in the two genes could therefore have additive or interacting effects on shared downstream pathways.

However, because *ADIPOQ* (chromosome 3q27) and *FNDC5* (chromosome 1p35) are located on different chromosomes, the two loci are not in linkage disequilibrium, and the allelic combinations analyzed here do not represent haplotypes inherited as a unit. Their frequencies were statistically estimated from unphased genotype data; therefore, this analysis should be regarded as an exploratory evaluation of the joint allele distribution rather than as evidence of a biological gene–gene interaction. For this reason, a genotype-based interaction analysis was additionally performed (Section 2.8).

The results of our combined two-locus allelic analysis should therefore be interpreted cautiously. In the overall PTB analysis, none of the investigated allelic combinations showed a significant association with PTB. The G–T allelic combination of *ADIPOQ* rs266729–*FNDC5* rs1746661 occurred in 6.6% of PTB cases compared with 4.6% of term controls and was not significantly associated with overall PTB (OR 1.45, 95% CI 0.70–3.01; *p* = 0.32).

However, in the early PTB subgroup, the G–T allelic combination was observed in 13.6% of early PTB cases compared with 4.6% of term controls. The unadjusted analysis yielded an OR of 2.83 (95% CI, 1.11–7.22; *p* = 0.03). This finding should be interpreted in light of the fact that the same subgroup also showed a borderline tendency for the *ADIPOQ* rs266729 G allele, so the two observations are not independent.

Nevertheless, statistical interpretation is crucial. The global allelic combination association was not significant (*p* = 0.10), and the association of the G–T allelic combination did not remain statistically significant after Benjamini–Hochberg correction (adjusted *p* = 0.09). Therefore, these findings should be regarded as exploratory rather than evidence of a confirmed association. Furthermore, an additional genotype-based interaction analysis did not identify a statistically significant interaction between *ADIPOQ* rs266729 and *FNDC5* rs1746661 (*p* = 0.177), although smaller interaction effects cannot be excluded.

A more cautious interpretation is that the two pathways may represent partially convergent metabolic and placental mechanisms, while the effect of any individual common variant is small. This is consistent with the multifactorial nature of PTB, in which several genetic variants of modest effect may interact with maternal metabolic state, inflammation, placental function, and environmental exposures [23,25].

### 3.4. Gestational-Age-Specific Effects and Clinical Phenotype

An important aspect of the present study is the decision to distinguish among early, extremely early, and late PTB rather than analyze all preterm deliveries as a single outcome. In this approach, a borderline, non-significant tendency was observed only in early PTB, whereas neither extremely early nor late PTB showed evidence of association.

The absence of an association in extremely early PTB should be interpreted in the context of the small subgroup size. Only 17 women were classified as having extremely early PTB, resulting in few individual genotypes and very wide confidence intervals. Consequently, the negative findings in this subgroup should not be interpreted as definitive evidence that the investigated variants have no role in extremely early delivery.

At the same time, the absence of an association in late PTB provides some support for the possibility that the *ADIPOQ* signal is not simply a reflection of a general tendency toward shorter gestation. In late PTB, the *ADIPOQ* G allele and GG genotype were not significantly associated with the outcome, and no significant allelic combination association was observed.

Thus, the most appropriate interpretation is that our data neither demonstrate a general genetic predisposition to PTB nor establish phenotype-specific susceptibility but raise a hypothesis that should be tested in adequately powered studies [24,25]. This distinction may be important for future genetic studies, particularly because combining biologically heterogeneous PTB phenotypes can obscure modest associations.

### 3.5. Strengths and Limitations

Several limitations should be acknowledged.

First, although the overall cohort provided a reasonable sample size for the exploratory candidate-gene analysis, stratification into early and extremely early PTB substantially reduced statistical power. This is particularly relevant for the extremely early PTB subgroup, where rare genotypes were represented by very small numbers.

The sample size was determined by the number of available eligible participants rather than by an a priori power calculation. A sensitivity power analysis indicated that, under the dominant inheritance model of *ADIPOQ* rs266729, the minimum detectable odds ratios were approximately 1.76 for overall PTB, 3.06 for early PTB, and 4.53 for extremely early PTB, assuming 80% power and a two-sided significance level of 0.05. These findings indicate that smaller genetic associations may have remained undetected, particularly in the gestational-age subgroups.

Second, for *ADIPOQ* rs266729, only a borderline difference in allele frequency was observed in early PTB (*p* = 0.05, with a 95% CI including 1), which was not supported by the genotype comparison, any inheritance model, or logistic regression. Therefore, no association between rs266729 and early PTB can be claimed on the basis of the present data.

Third, the nominal association observed for the G–T allelic combination did not survive correction for multiple comparisons. Moreover, the allelic combinations involve two unlinked loci on different chromosomes and were estimated from unphased genotypes, and the interaction analysis had limited power to detect small interaction effects and could not be performed in the gestational-age subgroups. In addition, numerous comparisons were performed across variants, inheritance models, and PTB subgroups without study-wide correction, which increases the risk of false-positive findings. Conversely, the limited power of the subgroup analyses increases the risk of false-negative results; thus, the absence of associations in this study should not be interpreted as evidence of no effect.

Fourth, only two variants were investigated, and therefore the study cannot exclude associations involving other functional variants within *ADIPOQ* or *FNDC5*.

Another limitation is that we investigated maternal genetic variation only. Maternal genotype represents only one component of the genetic architecture of PTB, and fetal genotype, maternal–fetal genetic interactions, and fetal/placental genetic effects were not assessed. In addition, smoking was recorded only as a binary variable; therefore, the potential effect of smoking intensity could not be assessed.

Furthermore, circulating adiponectin and irisin concentrations and placental expression of *ADIPOQ* and *FNDC5* were not measured. Consequently, the functional consequences of the investigated variants cannot be directly demonstrated in the present cohort. Likewise, no functional validation (e.g., reporter-gene assays or expression analyses) was performed; therefore, the mechanisms proposed in the Discussion remain hypothetical.

Despite these limitations, the study has several strengths.

The cohort was well characterized, maternal rather than fetal genetic effects were specifically investigated, multiple inheritance models were assessed, and Combined Two-Locus Allelic analyses were performed.

Importantly, PTB was also examined according to gestational age, allowing the exploration of potentially phenotype-specific effects that might be masked in the overall PTB analysis.

## 4. Materials and Methods

### 4.1. Study Subjects

This case–control study included 401 pregnant women who delivered at the Department of Obstetrics and Gynecology, University Hospital Centre Osijek, Croatia, between November 2017 and March 2024. The study population consisted of 200 women with preterm birth (PTB) and 201 women with term delivery, who served as the control group. None of the mothers in either group were genetically related. All available women who met the inclusion criteria were included in the study. No formal a priori sample size calculation was performed.

The control group included women who delivered single newborns between 37 and 41 + 3 weeks of gestation. If the pregnancy was uncomplicated, all deliveries were spontaneous. Women in the control group were selected to be comparable with the PTB group with respect to age, parity, socioeconomic and demographic characteristics, ethnicity, place of residence, antenatal care, and year of delivery.

Exclusion criteria for the control group: positive personal and/or family history of preterm birth, history of any cervical surgical procedure before or during pregnancy (e.g., conization, cerclage). At the end, mothers with confirmed or acute infections during labor were excluded based on clinical presentation, elevated inflammatory markers, and positive vaginal or cervical cultures.

The inclusion criterion for pregnant women in the preterm labor group was the birth of a single live newborn before 37 weeks of gestation with spontaneous onset of labor, with or without prelabor rupture of membranes (PROM) and hospital admission after the onset of labor.

The exclusion criteria for the preterm labor group were known risk factors for preterm birth (such as in vitro fertilization, multiple pregnancy, cervical surgery, inflammation of the reproductive organs and kidney disease), gestational diabetes mellitus, hypertension in pregnancy, signs of infection in laboratory tests shortly before birth, or signs of infection in the pathohistological analysis of the placenta.

Preterm birth was defined as delivery before 37 + 0 completed weeks of gestation, according to the World Health Organization definition, whereas term delivery was defined as birth at or after 37 + 0 weeks of gestation [1]. The group of pregnant women with premature birth was, for further analysis, subdivided into three subgroups by gestational age: extremely early PTB group (24–27 + 6 weeks of gestation, *n* = 17), early PTB group (28–31 + 6 weeks of gestation, *n* = 31), and late PTB group (32–36 + 6 weeks of gestation, *n* = 150). Gestational age at delivery was unavailable for two women with PTB; therefore, 198 women were included in the gestational age subgroup analyses [1].

Gestational age was determined based on first-trimester ultrasonography or, when unavailable, according to the first day of the last menstrual period. In the case of a discrepancy between the due date based on the first day of the last menstrual period and the ultrasound finding, gestational age was corrected according to the ultrasound finding [33].

### 4.2. Clinical Data Collection

Maternal demographic and clinical characteristics were obtained from medical records and standardized clinical documentation. The collected variables included maternal age, body mass index (BMI), smoking status, coffee consumption, prelabor rupture of membranes (PROM), personal and family history of PTB, gestational age at delivery, mode of delivery, and neonatal birth weight. Clinical and pregnancy-related risk factors were defined as the presence of at least one of the following: urinary tract infection, positive cervical microbiological findings, vaginal bleeding during pregnancy, or previous cervical conization.

Body mass index was calculated as weight (kg) divided by height squared (m^2^). Gestational age at delivery was recorded as completed weeks of gestation.

### 4.3. DNA Isolation and SNP Genotyping

Blood was collected from the mothers after admission to the delivery room, during the first stage of labor. For the analysis of genetic variants, 3 mL of the maternal venous blood was collected in vacuum tubes (Vacutainer, Becton Dickinson, Franklin Lakes, NJ, USA) containing the anticoagulant ethylenediaminetetraacetic acid (EDTA).

Two maternal genetic variants were selected for analysis: *ADIPOQ* rs266729 and *FNDC5* rs1746661. The selection was based on their potential biological relevance to metabolic regulation and previously reported associations with metabolic and pregnancy-related phenotypes. The functional and regulatory potential of the variants was assessed using RegulomeDB [19] and the GTEx portal [21]. *ADIPOQ* rs266729 (−11377C>G) is located in the promoter region; has a RegulomeDB rank of 1f (eQTL evidence combined with transcription factor binding or a DNase I hypersensitivity peak) [19,20], although it is not reported as a significant eQTL for *ADIPOQ* in GTEx [21]; has been associated with circulating adiponectin concentrations [18]; and has been studied in relation to GDM [8,9]. *FNDC5* rs1746661 is an intronic variant that has been investigated in relation to insulin sensitivity, glucose and lipid metabolism, and blood pressure [34,35] and is one of only two *FNDC5* variants previously examined in relation to PTB [14]; it has a RegulomeDB rank of 1b (probability score 0.97), indicating eQTL evidence together with transcription factor binding, a transcription factor motif, a DNase footprint, and a DNase I hypersensitivity peak [19]; and in GTEx, it is a significant eQTL for *FNDC5* in skeletal muscle (*p* = 1.9 × 10^−57^) [21]. Neither variant is a non-synonymous SNP. The characteristics of both variants are summarized in Table 8. The variants were analyzed in maternal genomic DNA. Genotype analyses were performed using all available valid genotype data. Samples for which a valid genotype call was not available were excluded from the respective genetic analysis; therefore, the number of participants differed slightly between the two variants.

Genomic deoxyribonucleic acid (DNA) was extracted from 200 µL of EDTA-anticoagulated whole blood using commercially available spin columns QIAamp DNA Blood Mini Kit (Qiagen GmbH, Hilden, Germany), according to the manufacturer’s instructions [38]. DNA samples were stored at −20 °C until further analysis. The quality and purity of the isolated DNA were assessed using a Qubit 3 fluorometer (Thermo Fisher Scientific, Waltham, MA, USA).

SNP genotyping was performed using TaqMan-based fluorescent probes (TaqMan SNP Genotyping Assays, Waltham, MA, USA) on an ABI PRISM 7500 real-time polymerase chain reaction (PCR) system (Applied Biosystems, Foster City, CA, USA) [39]. The thermocycling procedure was as follows: 1 hold at 95 °C for 10 min; 40 cycles of denaturation at 92 °C for 15 s and primer annealing and extension at 60 °C for 1 min. Negative and positive control samples were run simultaneously on each real-time PCR plate. The total reaction volume per well was 25 µL, with 2 µL of DNA used as a template. Allelic discrimination analysis was performed using SDS 7500 Software Version 2.3 (Applied Biosystems, Foster City, CA, USA).

Genotype assignment was based on automated analysis of allelic discrimination data, followed by manual inspection of the allelic discrimination plots. Genotyping quality was assessed separately for each assay based on the genotype call rate and between-run replicate concordance (Table S7: Genotyping quality control parameters for *ADIPOQ* rs266729 and *FNDC5* rs1746661). Initially unsuccessful reactions (*n* = 61 across both assays) were repeated, and the final call rate was 99.25% for *ADIPOQ* rs266729 (398/401 samples) and 98.00% for *FNDC5* rs1746661 (393/401 samples). To assess reproducibility, 54 samples (rs266729) and 66 samples (rs1746661) were re-analyzed in independent qPCR runs, with 100% concordance between the original and repeated genotype calls. Only samples with valid genotype calls were included in the statistical analyses.

### 4.4. Statistical Analysis

Categorical data were presented as absolute and relative frequencies, and continuous data as medians and interquartile ranges (IQRs). Differences between groups were assessed using the chi-square test or Fisher’s exact test for categorical variables and the Mann–Whitney U test for continuous variables.

Genotype and allele frequencies were compared between groups, and Hardy–Weinberg equilibrium was assessed. Associations of *ADIPOQ* rs266729 and *FNDC5* rs1746661 with preterm birth were evaluated under dominant, recessive, and overdominant inheritance models and expressed as odds ratios (ORs) with 95% confidence intervals (CIs). Combined two-locus allelic analyses were additionally performed. Genetic analyses were conducted using the SNPStats web tool (https://www.snpstats.net/start.htm (accessed on 7 May 2025), Solé et al., 2006) [40].

Bivariate logistic regression was used to assess associations between the investigated genetic variants and preterm birth. For multiple comparisons of allelic combinations, *p* values were adjusted using the Benjamini–Hochberg procedure to control the false discovery rate at 5%. Because *ADIPOQ* and *FNDC5* are located on different chromosomes, allelic combination frequencies were estimated from unphased genotype data using the expectation–maximization algorithm implemented in SNPStats and were interpreted as exploratory. To complement this approach, a genotype-based gene–gene interaction analysis was performed using binary logistic regression with both variants coded under the dominant model and their product term; model fit was assessed with the omnibus chi-square test and Nagelkerke R^2^. No correction for multiple testing was applied across variants, inheritance models, and PTB subgroups; these results are therefore reported as nominal. Analyses stratified by gestational age were considered exploratory due to the relatively small subgroup sizes.

No formal a priori sample size calculation was performed, as all available women meeting the inclusion criteria were included in the study. A sensitivity power analysis was performed using G*Power 3.1.9.7 to estimate the minimum detectable difference between two independent proportions, assuming a two-sided significance level of 0.05 and 80% statistical power. The analysis was based on the observed carrier frequency in the term-delivery group under the dominant inheritance model of *ADIPOQ* rs266729 and did not account for multiple-comparison correction.

Statistical analyses were performed using MedCalc^®^ Statistical Software version 23.7 (MedCalc Software Ltd., Ostend, Belgium; 2026). All tests were two-sided, with the level of statistical significance set at α = 0.05.

## 5. Conclusions

In summary, the present study did not identify statistically significant associations between maternal *ADIPOQ* rs266729 or *FNDC5* rs1746661 variants and overall spontaneous preterm birth. No significant gene–gene interaction between the two variants was observed. Although a borderline tendency toward a higher *ADIPOQ* rs266729 G allele frequency was observed among women with early PTB, without a significant difference in GG genotype frequency, and a nominal association was found for the *ADIPOQ–FNDC5* G–T allelic combination, the latter did not remain statistically significant after correction for multiple comparisons. Given the small subgroup sizes and the exploratory nature of these analyses, these findings should be interpreted with caution and do not provide sufficient evidence for an association between the investigated genetic variants and specific PTB phenotypes.

The absence of a significant association for *FNDC5* rs1746661, despite experimental evidence implicating the *FNDC5*/irisin pathway in placental function, highlights the complexity of translating biological mechanisms into detectable associations with individual common genetic variants. The observed findings should therefore be considered exploratory and hypothesis-generating rather than confirmatory.

Future studies should investigate larger and ethnically diverse cohorts and integrate maternal genetic data with circulating adiponectin and irisin concentrations, maternal metabolic phenotype, inflammatory biomarkers, placental gene expression, and detailed gestational-age-specific PTB phenotyping. Such an integrated approach may clarify whether variation in metabolic endocrine pathways contributes to the timing of spontaneous parturition and whether the borderline *ADIPOQ* tendency observed in early PTB represents a reproducible biological association.

## Figures and Tables

**Table 1:** Maternal and neonatal characteristics and clinical and pregnancy-related risk factors in women with term and preterm birth.

	Term Birth (*n* = 201)	PTB (*n* = 200)	Total (*n* = 401)	*p* Value
Mother’s age (years) [Median (IQR)]	30 (26–34)	31 (27–35)	31 (27–35)	0.20 ^‡^
BMI (kg/m^2^) [Median (IQR)]	27.4 (24.5–30.7)	26.7 (24.2–30.1)		0.30 ^‡^
Underweight [*n* (%)]	2 (1)	0	2 (0.5)	0.15 ^†^
Normal weight [*n* (%)]	54 (26.9)	66 (33)	120 (29.9)	
Overweight [*n* (%)]	145 (72.1)	134 (67)	279 (69.6)	
Coffee consumption [*n* (%)]	168 (83.6)	156 (78.0)	324 (80.8)	0.16 *
Smoking habit [*n* (%)]	52 (25.9)	63 (31.5)	115 (28.7)	0.21 *
Clinical and pregnancy-related risk factors [*n* (%)]	104 (51.7)	151 (75.5)	255 (63.6)	**<0.001** *
Urinary tract infection [*n* (%)]	18 (9.0)	27 (13.5)	45 (11.2)	0.15 *
Positive cervical swabs [*n* (%)]	44 (21.9)	35 (17.5)	79 (19.7)	0.27 *
Vaginal bleeding during pregnancy [*n* (%)]	14 (7.0)	46 (23.0)	60 (15.0)	**<0.001** *
PROM [*n* (%)]	47 (23.4)	118 (59.6)	165 (41.4)	**<0.001** *
Family history of PTB [***n*** (%)]	0	33 (16.5)	33 (8.2)	**<0.001** *
History of previous PTB [*n* (%)]	0	30 (15)	30 (7.5)	**<0.001** *
Number of previous PTB among women with a history of PTB [Median (min–max)]	-	1 (1–3)	-	-
Preterm birth in personal anamnesis [*n* (%)]				
Extremely early PTB	-	8 (26.7)	-	-
Early PTB	-	4 (13.3)	-	-
Late PTB	-	22 (73.3)	-	-
Number of previous births [Median (IQR)]	2 (1–2)	1 (1–2)	-	0.25 ^‡^
Gestational age [Median (IQR)]	39 + 4 (39 + 0–40 + 3)	34 + 6 (32 + 0–36 + 1)		**<0.001 ^‡^**
Mode of delivery [*n* (%)]				
Vaginal birth	196 (97.5)	142 (71)	338 (84.3)	**<0.001** *
Cesarean section	5 (2.5)	58 (29)	63 (15.7)	
Birth weight of infants [g]	3450 (3125–3800)	2425 (1797–2780)		**<0.001 ^‡^**

* Chi-square test; ^†^ Fisher’s exact test; ^‡^ Mann–Whitney U test. Abbreviations: BMI, body mass index; IQR, interquartile range; PROM, prelabor rupture of membranes; PTB, preterm birth. Due to missing data, percentages were calculated based on available observations. Categories of previous preterm births were not mutually exclusive, as women could have experienced preterm births at different gestational ages; therefore, percentages may sum to more than 100%. Personal and family history of PTB were exclusion criteria for the control group. Bold denotes statistical significance.

**Table 2:** Genotype distribution and allele frequencies of two selected SNPs of *ADIPOQ* and *FNDC5* in mothers with preterm birth and respective controls.

SNP/Inheritance Model		Genotype [*n* (%)]		OR (95% CI)	*p* * Value
		Term Birth	PTB		
^a^ *ADIPOQ* (rs266729)		*n* = 199	*n* = 199		
	C/C	108 (54.3)	93 (46.7)	1.0	0.27
	C/G	76 (38.2)	85 (42.7)	1.30 (0.86–1.97)	
	G/G	15 (7.5)	21 (10.6)	1.63 (0.79–3.33)	
Alleles	C	292 (73)	271 (68)	1.29 (0.95–1.75)	0.10
	G	106 (27)	127 (32)		
Dominant inheritance model	C/C	108 (54.3)	93 (46.7)	1.0	0.13
	C/G–G/G	91 (45.7)	106 (53.3)	1.35 (0.91–2.01)	
Recessive inheritance model	C/C–C/G	184 (92.5)	178 (89.5)	1.0	0.29
	G/G	15 (7.5)	21 (10.6)	1.45 (0.72–2.90)	
Overdominant inheritance model	C/C–G/G	123 (61.8)	114 (57.3)	1.0	0.36
	C/G	76 (38.2)	85 (42.7)	1.21 (0.81–1.80)	
^b^ *FNDC5* (rs1746661)		*n* = 198	*n* = 195		
	G/G	122 (61.6)	133 (68.2)	1.0	0.39
	G/T	68 (34.3)	56 (28.7)	0.76 (0.49–1.16)	
	T/T	8 (4)	6 (3.1)	0.69 (0.23–2.04)	
Alleles	G	312 (79)	322 (83)	0.78 (0.55–1.12)	0.18
	T	84 (21)	68 (17)		
Dominant inheritance model	G/G	122 (61.6)	133 (68.2)	1.0	0.17
	G/T–T/T	76 (38.4)	62 (31.8)	0.75 (0.49–1.13)	
Recessive inheritance model	G/G–G/T	190 (96)	189 (96.9)	1.0	0.61
	T/T	8 (4)	6 (3.1)	0.75 (0.26–2.21)	
Overdominant inheritance model	G/G–T/T	130 (65.7)	139 (71.3)	1.0	0.23
	G/T	68 (34.3)	56 (28.7)	0.77 (0.50–1.18)	

* Chi-squared test. Abbreviations: SNP, single-nucleotide polymorphism; PTB, preterm birth; OR, odds ratio; CI, confidence interval; *ADIPOQ*, adiponectin C1Q and collagen domain containing gene; *FNDC5*, fibronectin type III domain-containing protein 5 gene. ^a^—Hardy–Weinberg equilibrium: Term Birth *p* = 0.72; PTB *p* = 0.87; ^b^—Hardy–Weinberg equilibrium: Term Birth *p* = 0.83; PTB *p* > 0.99.

**Table 3:** Allelic combinations of rs266729 and rs1746661 and their association with preterm birth.

Allelic Combinations		Allelic Combination Frequency (%)		OR (95% CI)	*p* *	*_adj_p*
rs266729	rs1746661	Term Birth	PTB			
C	G	56.7	57.3	1.0	-	-
G	G	22.0	25.3	1.11 (0.77–1.61)	0.57	0.57
C	T	16.6	10.8	0.62 (0.37–1.03)	0.06	0.19
G	T	4.6	6.6	1.45 (0.70–3.01)	0.32	0.48

* Unadjusted *p* value; *_adj_p* value adjusted for multiple comparisons using the Benjamini–Hochberg method; OR, odds ratio; CI, confidence interval; PTB, preterm birth. Global allelic combination association *p* = 0.10.

**Table 4:** Prediction of preterm birth using bivariate logistic regression analysis.

Bivariate Regression	β	Wald	*p* Value	OR (95% CI)
rs266729 (C/G + G/G vs. C/C)	0.30	2.26	0.13	1.35 (0.92–2.01)
rs1746661 (G/T + T/T vs. G/G)	−0.29	1.87	0.17	0.75 (0.49–1.13)

β, regression coefficient; OR, odds ratio; CI, confidence interval.

**Table 5:** Genotype distribution and allele frequencies of two selected SNPs of *ADIPOQ* and *FNDC5* in mothers with early preterm birth and respective controls.

SNP/Inheritance Model		Genotype [*n* (%)]		OR (95% CI)	*p* * Value
		Term Birth	Early PTB		
^a^ *ADIPOQ* (rs266729)		*n* = 199	*n* = 31		
	C/C	108 (54.3)	12 (38.7)	1.0	0.17
	C/G	76 (38.2)	14 (45.2)	1.66 (0.73–3.78)	
	G/G	15 (7.5)	5 (16.1)	3.00 (0.93–9.71)	
Alleles	C	292 (73)	38 (61)	1.74 (0.99–3.04)	0.05
	G	106 (27)	24 (39)		
Dominant inheritance model	C/C	108 (54.3)	12 (38.7)	1.0	0.11
	C/G–G/G	91 (45.7)	19 (61.3)	1.88 (0.87–4.08)	
Recessive inheritance model	C/C–C/G	184 (92.5)	26 (83.9)	1.0	0.15
	G/G	15 (7.5)	5 (16.1)	2.36 (0.79–7.03)	
Overdominant inheritance model	C/C–G/G	123 (61.8)	17 (54.8)	1.0	0.46
	C/G	76 (38.2)	14 (45.2)	1.33 (0.62–2.86)	
^b^ *FNDC5* (rs1746661)		*n* = 198	*n* = 29		
	G/G	122 (61.6)	18 (62.1)	1.0	0.78
	G/T	68 (34.3)	9 (31)	0.90 (0.38–2.11)	
	T/T	8 (4)	2 (6.9)	1.69 (0.33–8.62)	
Alleles	G	312 (79)	45 (78)	1.07 (0.55–2.08)	0.83
	T	84 (21)	13 (22)		
Dominant inheritance model	G/G	122 (61.6)	18 (62.1)	1.0	0.96
	G/T–T/T	76 (38.4)	11 (37.9)	0.98 (0.44–2.19)	
Recessive inheritance model	G/G–G/T	190 (96)	27 (93.1)	1.0	0.51
	T/T	8 (4)	2 (6.9)	1.76 (0.35–8.72)	
Overdominant inheritance model	G/G–T/T	130 (65.7)	20 (69)	1.0	0.72
	G/T	68 (34.3)	9 (31)	0.86 (0.37–1.99)	

* Chi-squared test. Abbreviations: SNP, single-nucleotide polymorphism; PTB, preterm birth; OR, odds ratio; CI, confidence interval; *ADIPOQ*, adiponectin C1Q and collagen domain containing gene; *FNDC5*, fibronectin type III domain-containing protein 5 gene. ^a^—Hardy–Weinberg equilibrium: Term Birth *p* = 0.73; PTB *p* = 0.72; ^b^—Hardy–Weinberg equilibrium: Term Birth *p* = 0.84; PTB *p* = 0.83.

**Table 6:** Allelic combinations of rs266729 and rs1746661 and their association with early preterm birth.

Allelic Combination		Allelic Combination Frequency (%)		OR (95% CI)	*p* *	_adj_ *p* ^†^
rs266729	rs1746661	Term Birth	EarlyPTB			
C	G	56.8	52.7	1.0	-	
G	G	22.0	25.1	1.20 (0.59–2.44)	0.61	0.61
C	T	16.6	8.6	0.54 (0.17–1.72)	0.30	0.45
G	T	4.6	13.6	2.83 (1.11–7.22)	**0.03**	0.09

* Unadjusted *p* value; ^†^ *_adj_p* value adjusted for multiple comparisons using the Benjamini–Hochberg method; OR, odds ratio; CI, confidence interval; PTB, preterm birth. Global allelic combination association *p* = 0.10. Bold denotes statistical significance.

**Table 7:** Prediction of early preterm birth using bivariate logistic regression analysis.

Bivariate Regression	β	Wald	*p* Value	OR (95% CI)
rs266729 (C/G + G/G vs. C/C)	0.52	1.84	0.18	1.68 (0.79–3.56)
rs1746661 (G/T + T/T vs. G/G)	0.13	0.11	0.74	1.14 (0.52–2.49)

β, regression coefficient; OR, odds ratio; CI, confidence interval.

**Table 8:** Studied *ADIPOQ* and *FNDC5* single-nucleotide polymorphisms (SNPs).

SNP	Location	Gene	Gene Region	Base Change	Citation	Related Phenotype
**rs266729**	chr3:186841685 (GRCh38.p14)	***ADIPOQ***	Promoter region (RegulomeDB rank 1f)	C/G	Hsiao and Lin [36]; Al Saleh et al. [37]	Obesity; BMI; metabolic phenotypes
**rs1746661**	chr1:32869438 (GRCh38.p14)	***FNDC5***	Intron 2 (RegulomeDB rank 1b)	G/T	Staiger et al. [34]; Al-Daghri et al. [35]	Insulin sensitivity; glucose and lipid metabolism; blood pressure

Abbreviations: *ADIPOQ*, adiponectin C1Q and collagen domain containing gene; *FNDC5*, fibronectin type III domain-containing protein 5 gene.

## Data Availability

Dataset available on request from the authors. The raw data supporting the conclusions of this article will be made available by the authors on request.

## Supplementary Materials

The following supporting information can be downloaded at: https://www.mdpi.com/article/10.3390/ijms27219945/s1.

## Abbreviations

The following abbreviations are used in this manuscript:

*ADIPOQ*	adiponectin, C1Q and collagen domain containing
AMPK	AMP-activated protein kinase
BH	Benjamini–Hochberg
BMI	body mass index
CI	confidence interval
eQTL	expression quantitative trait locus
*FNDC5*	fibronectin type III domain-containing protein 5
GA	gestational age
GDM	gestational diabetes mellitus
GTEx	Genotype-Tissue Expression
HWE	Hardy–Weinberg equilibrium
LD	linkage disequilibrium
OR	odds ratio
PROM	prelabor rupture of membranes
PTB	preterm birth
qPCR	quantitative real-time polymerase chain reaction
SNP	single-nucleotide polymorphism
