# Supplementary material for: Maternal FNDC5 and ADIPOQ Genetic Variants as Potential Modulators of Susceptibility to Spontaneous Preterm Birth
Source: Int J Mol Sci. 2026 Nov 6;27(21):9945. doi: 10.3390/ijms27219945 (PMC13763604; doi:10.3390/ijms27219945)
Supplement: Supplementary file 1 [file ijms-27-09945-s001.zip › ijms-4644842-supplementary.pdf]

**Table S1** Genotype distribution and allele frequencies of two selected SNPs of *ADIPOQ* and *FNDC5* in mothers with extremely early preterm birth and respective controls

| SNP/Inheritance model | | Genotype [n (%)] | | OR (95 % CI) | *P** value |
|---|---|---|---|---|---|
| | | Term birth | Extremely early PTB | | |
| **[a]*ADIPOQ* (rs266729)** | | **n=199** | **n=17** | | |
| | C/C | 108 (54.3) | 10 (58.8) | 1.0 | |
| | C/G | 76 (38.2) | 6 (35.3) | 0.85 (0.30-2.45) | 0.93 |
| | G/G | 15 (7.5) | 1 (5.9) | 0.72 (0.09-6.03) | |
| Alleles | C | 292 (73) | 26 (76) | 0.85 (0.37-1.93) | 0.69 |
| | G | 106 (27) | 8 (24) | | |
| Dominant inheritance model | C/C | 108 (54.3) | 10 (58.8) | 1.0 | 0.72 |
| | C/G–G/G | 91 (45.7) | 7 (41.2) | 0.83 (0.30-2.27) | |
| Recessive inheritance model | C/C–C/G | 184 (92.5) | 16 (94.1) | 1.0 | 0.80 |
| | G/G | 15 (7.5) | 1 (5.9) | 0.77 (0.10-6.18) | |
| Overdominant inheritance model | C/C–G/G | 123 (61.8) | 11 (64.7) | 1.0 | 0.81 |
| | C/G | 76 (38.2) | 6 (35.3) | 0.88 (0.31-2.49) | |
| **[a]*FNDC5* (rs1746661)** | | **n=198** | **n=17** | | |
| | G/G | 122 (61.6) | 14 (82.3) | 1.0 | |
| | G/T | 68 (34.3) | 3 (17.6) | 0.38 (0.11-1.39) | 0.14 |
| | T/T | 8 (4) | 0 | 0.0 (0.0-NA) | |
| Alleles | G | 312 (79) | 31 (91) | 0.36 (0.11-1.20) | 0.10 |
| | T | 84 (21) | 3 (9) | | |
| Dominant inheritance model | G/G | 122 (61.6) | 14 (82.3) | 1.0 | 0.07 |
| | G/T–T/T | 76 (38.4) | 3 (17.6) | 0.34 (0.10-1.24) | |
| Recessive inheritance model | G/G–G/T | 190 (96) | 17 (100) | 1.0 | 0.25 |
| | T/T | 8 (4) | 0 | 0.0 (0.0-NA) | |
| Overdominant inheritance model | G/G–T/T | 130 (65.7) | 14 (82.3) | 1.0 | 0.14 |
| | G/T | 68 (34.3) | 3 (17.6) | 0.41 (0.11-1.47) | |

* Chi-squared test. Abbreviations: SNP, single nucleotide polymorphism; PTB, preterm birth; OR, odds ratio; CI, confidence interval; *ADIPOQ*, adiponectin C1Q and collagen domain containing gene; *FNDC5*, fibronectin type III domain-containing protein 5 gene.
a - Hardy-Weinberg equilibrium: Term Birth *P*=0.73; PTB *P*=0.72
b - Hardy-Weinberg equilibrium: Term Birth *P*=0.84; PTB *P*=0.83

Table S2. Allelic combination frequencies of rs266729 and rs1746661 and their association with extremely early preterm birth

| Allelic combinations | | Allelic combination frequency (%) | | OR (95 % CI) | P* | $_{adj}P^{†}$ |
|---|---|---|---|---|---|---|
| rs266729 | rs1746661 | Term birth | Extremely early PTB | | | |
| C | G | 57.8 | 67.7 | 1.0 | - | |
| G | G | 22.1 | 23.5 | 0.86 (0.33-2.25) | 0.75 | 0.76 |
| C | T | 15.9 | 8.8 | 0.40 (0.08-2.07) | 0.28 | 0.76 |

| G | T | 4.3 | 0.0 | 0.09 (0.0-414047.5) | 0.76 | 0.76 |
|---|---|---|---|---|---|---|

*Unadjusted *P* value; †*P* value adjusted for multiple comparisons using the Benjamini–Hochberg method; OR, odds ratio; CI, confidence interval; PTB, preterm birth. Global allelic combination association *P* = 0.26.

Table S3. Prediction of extremely early preterm birth using bivariate logistic regression analysis

| Bivariate regression | β | Wald | *P value* | OR (95% CI) |
|---|---|---|---|---|
| rs266729 (C/G + G/G vs. C/C ) | -0.35 | 0.49 | 0.49 | 0.70 (0.26-1.89) |
| rs1746661 (G/T + T/T vs. G/G) | -0.96 | 2.22 | 0.14 | 0.38 (0.11-1.36) |

β, regression coefficient; OR, odds ratio; CI, confidence interval

**Table S4.** Genotype distribution and allele frequencies of two selected SNPs of *ADIPOQ* and *FNDC5* in mothers with late preterm birth and respective controls

| SNP/Inheritance model | | Genotype [n (%)] | | OR (95 % CI) | *P** value |
|---|---|---|---|---|---|
| | | Term birth | Late PTB | | |
| [a]***ADIPOQ* (rs266729)** | | **n=199** | **n=149** | | |
| | C/C | 108 (54.3) | 70 (47) | 1.0 | |
| | C/G | 76 (38.2) | 64 (43) | 1.30 (0.83-2.03) | 0.37 |
| | G/G | 15 (7.5) | 15 (10.1) | 1.54 (0.71-3.35) | |
| Alleles | C | 292 (73) | 204 (68) | 1.26 (0.91-1.77) | 0.16 |
| | G | 106 (27) | 94 (32) | | |
| Dominant inheritance model | C/C | 108 (54.3) | 70 (47) | 1.0 | 0.18 |
| | C/G–G/G | 91 (45.7) | 79 (53) | 1.34 (0.87-2.05) | |
| Recessive inheritance model | C/C–C/G | 184 (92.5) | 134 (89.9) | 1.0 | 0.41 |
| | G/G | 15 (7.5) | 15 (10.1) | 1.37 (0.65-2.91) | |
| Overdominant inheritance model | C/C–G/G | 123 (61.8) | 85 (57) | 1.0 | 0.37 |
| | C/G | 76 (38.2) | 64 (43) | 1.22 (0.79-1.88) | |
| [a]***FNDC5* (rs1746661)** | | **n=198** | **n=147** | | |
| | G/G | 122 (61.6) | 99 (67.3) | 1.0 | 0.50 |
| | G/T | 68 (34.3) | 44 (29.9) | 0.80 (0.50-1.27) | |

| | | | | | |
|---|---|---|---|---|---|
| | T/T | 8 (4) | 4 (2.7) | 0.62 (0.18-2.11) | |
| Alleles | G | 312 (79) | 242 (82) | 0.80 (0.54-1.17) | 0.25 |
| | T | 84 (21) | 52 (18) | | |
| Dominant inheritance model | G/G | 122 (61.6) | 99 (67.3) | 1.0 | 0.27 |
| | G/T–T/T | 76 (38.4) | 48 (32.6) | 0.78 (0.50-1.22) | |
| Recessive inheritance model | G/G–G/T | 190 (96) | 143 (97.3) | 1.0 | 0.50 |
| | T/T | 8 (4) | 4 (2.7) | 0.66 (0.20-2.25) | |
| Overdominant inheritance model | G/G–T/T | 130 (65.7) | 103 (70.1) | 1.0 | 0.39 |
| | G/T | 68 (34.3) | 44 (29.9) | 0.82 (0.52-1.29) | |

* Chi-squared test. Abbreviations: SNP, single nucleotide polymorphism; PTB, preterm birth; OR, odds ratio; CI, confidence interval; *ADIPOQ*, adiponectin C1Q and collagen domain containing gene; *FNDC5*, fibronectin type III domain-containing protein 5 gene.

a - Hardy-Weinberg equilibrium: Term Birth *P*=0.73; PTB *P*=0.72

b - Hardy-Weinberg equilibrium: Term Birth *P*=0.84; PTB *P*=0.83

Table S5. Allelic combination frequencies of rs266729 and rs1746661 and their association with late preterm birth

| Allelic combinations | | Allelic combination frequency (%) | | OR (95 % CI) | P* | $_{adj}$P† |
|---|---|---|---|---|---|---|
| rs266729 | rs1746661 | Term birth | Late PTB | | | |
| C | G | 56.8 | 56.4 | 1.0 | | |
| G | G | 22.0 | 25.9 | 1.17 (0.78-1.74) | 0.45 | 0.60 |
| C | T | 16.6 | 12.1 | 0.71 (0.41-1.22) | 0.22 | 0.44 |
| G | T | 4.6 | 5.6 | 1.26 (0.54-2.90) | 0.60 | 0.60 |

*Unadjusted *P* value; †*P* value adjusted for multiple comparisons using the Benjamini–Hochberg method; OR, odds ratio; CI, confidence interval; PTB, preterm birth. Global allelic combination association *P* = 0.30.

Table S6. Prediction of late preterm birth using bivariate logistic regression analysis

| Bivariate regression | β | Wald | *P value* | OR (95% CI) |
|---|---|---|---|---|
| rs266729 (C/G + G/G vs. C/C ) | 0.23 | 1.18 | 0.28 | 1.25 (0.83-1.88) |
| rs1746661 (G/T + T/T vs. G/G) | -0.17 | 0.62 | 0.43 | 0.84 (0.55-1.29) |

β, regression coefficient; OR, odds ratio; CI, confidence interval

**Table S7.** Genotyping quality control parameters for *ADIPOQ* rs266729 and *FNDC5* rs1746661.

| Quality control parameter | *ADIPOQ* rs266729 | *FNDC5* rs1746661 |
|---|---|---|
| Total samples tested | 401 | 401 |
| Successful genotype calls | 398 | 393 |
| Call rate (%) | 99.25 | 98.00 |
| Samples without valid genotype | 3 | 8 |
| Samples retested for reproducibility | 54 | 66 |
| Replicate concordance (%) | 100 | 100 |

Call rate was calculated as the percentage of samples with a valid genotype call after completion of repeat testing, relative to the total number of samples tested. Reproducibility was assessed by re-analysis of samples in independent qPCR runs.
